# Multi-view united transformer block of graph attention network based autism spectrum disorder recognition

**DOI:** 10.3389/fpsyt.2025.1485286

**Published:** 2025-02-20

**Authors:** D. Darling Jemima, A. Grace Selvarani, J. Daphy Louis Lovenia

**Affiliations:** ^1^ Department of Computer Science and Engineering, Sri Krishna College of Technology, Coimbatore, India; ^2^ Department of Computer Science and Engineering, Sri Ramakrishna Engineering College, Coimbatore, India; ^3^ Department of Mathematics, Karunya Institute of Technology and Sciences, Coimbatore, India

**Keywords:** MVUT_GAT, transformer block, autism spectrum disorder, deep learning, neuroimaging

## Abstract

**Introduction:**

Autism Spectrum Disorder (ASD) identification poses significant challenges due to its multifaceted and diverse nature, necessitating early discovery for operative involvement. In a recent study, there has been a lot of talk about how deep learning algorithms might improve the diagnosis of ASD by analyzing neuroimaging data.

**Method:**

To overrule the negatives of current techniques, this research proposed a revolutionary strategic model called the Unified Transformer Block for Multi-View Graph Attention Networks (MVUT_GAT). For the purpose of extracting delicate outlines from physical and efficient functional MRI data, MVUT_GAT combines the advantages of multi-view learning with attention processes.

**Result:**

With the use of the ABIDE dataset, a thorough analysis shows that MVUT_GAT performs better than Mutli-view Site Graph Convolution Network (MVS_GCN), outperforming it in accuracy by +3.40%. This enhancement reinforces our suggested model’s effectiveness in identifying ASD. The result has implications over higher accuracy metrics. Through improving the accuracy and consistency of ASD diagnosis, MVUT_GAT will help with early interference and assistance for ASD patients.

**Discussion:**

Moreover, the proposed MVUT_GAT’s which patches the distance between the models of deep learning and medical visions by helping to identify biomarkers linked to ASD. In the end, this effort advances the knowledge of recognizing autism spectrum disorder along with the powerful ability to enhance results and the value of people who are undergone.

## 1 Introduction

Autism Spectrum Disorder (ASD) identification has emerged as a crucial issue within the field of neurodevelopmental research, as the prevalence of ASD grows worldwide. ASD is a complex and diverse neurodevelopmental syndrome marked by difficulties in social communication and repetitive activities. Identifying ASD early in a child’s life is critical for appropriate intervention and support, yet it remains a significant difficulty owing to the complex and frequently subtle nature of the symptoms. Traditional diagnostic procedures depend mainly on clinical observations, behavioral evaluations, and interviews, which are subjective, time-consuming, and may lack the sensitivity required for early diagnosis (1). In recent years, the use of deep learning approaches into ASD detection has showed great potential and drew major interest from researchers and clinicians. Deep learning, a form of machine learning that uses artificial neural networks modelled after the human brain, provides a unique technique to identify the detailed patterns and traits linked with ASD in neuroimaging data. Magnetic resonance imaging (MRI) and functional MRI (fMRI) have emerged as critical methods for understanding the brain’s anatomical and functional connections in a non-invasive manner (2). Deep learning methods, particularly neural networks with numerous layers, have shown the ability to automatically learn and extract nuanced patterns from neuroimaging data, allowing for the detection of minor anomalies linked with ASD. Minor anomalies are minor differences in the structure and function of the brain, undetectable using conventional methods and often too minor to be easily visible. Such minor differences include slight variations in the amygdala and cerebellum, abnormal connectivity patterns between areas of the brain, alterations in cortical thickness or volume, or microstructural changes in white matter. Advanced deep learning algorithms identify such minor differences when processing neuroimaging data. Thus, these minor differences are associated with some cognitive and behavioral features of ASD, which allows for earlier and more precise diagnosis. Deep learning has the ability to overcome various issues that standard approaches confront while identifying ASD. Deep learning algorithms can effectively handle large volumes of data, identifying nuanced patterns that would be difficult to detect manually. Furthermore, these models may incorporate multimodal information by incorporating data from a variety of sources, including structural and functional MRI, genetic markers, and behavioral evaluations. The end result is a more thorough and nuanced knowledge of ASD’s neurological roots, which might lead to more accurate and early detection. Furthermore, the incorporation of attention processes into deep learning models enables the prioritizing of key variables, improving interpretability and enabling the discovery of biomarkers linked with ASD. Understanding how deep learning models make decisions is crucial for bridging the gap between their complex, ‘black box’ nature and the clinical insights needed to make informed decisions in practice As deep learning advances, new architectures, optimization approaches, and hybrid models are being developed to improve the accuracy, resilience, and generalizability of ASD diagnosis models.

## 2 Related work

### 2.1 Recognizing ASD via various deep learning models

Functional Connectivity in ASD (3) is complex and shows hypo- and hyper-connectivity within many brain networks. Dimensional clustering can determine unique FC subtypes that relate to specific behavioral characteristics, hence unearthing a wide variety of connections between brain activity and behavior. Recognition of common patterns in FC would explain the heterogeneity of ASD better, facilitate better diagnosis, and develop tailored interventions that bridge neural changes to observable symptoms. The use of neuroimaging methods to better understand and diagnose neurodevelopmental disorders suggests a possible option for enhancing clinical practice. A technique for estimating effective connectivity in brain networks (BNs) (4) using EEG data, with a focus on children with attention-deficit hyperactivity disorder (ADHD). Their findings revealed substantial differences in directed information transmission across EEG electrodes in ADHD patients compared to healthy controls, with discriminative power notably high in the theta-band, which is associated with focus and motor activity. Similarly (5), used functional MRI (fMRI) data from the Autism Brain Imaging Data Exchange (ABIDE) dataset to solve the problem of multi-site data aggregation in autism diagnosis. They used data harmonization approaches to improve classification accuracy and discovered insights into ASD pathogenesis through network analysis. With machine learning techniques applied to fMRI functional connectivity, promising detection of ASD can be automated. The critical advances include integration of temporal dynamics, multiscale data, and focused analysis of brain networks relevant to ASD. Even though performance keeps improving, more work is needed to create robust and interpretable models for clinical application. Combining data modalities with larger datasets may further propel this field toward reliable computer-aided diagnosis of ASD (6). Their research produced encouraging results and revealed changes in brain network structure related to ASD. Furthermore (7), the significance of using sequential information from task-based fMRI for synthetic data augmentation in ASD diagnosis is demonstrated, demonstrating the potential for improving diagnostic accuracy and comprehending the underlying illness. Together, these studies highlight the value of neuroimaging techniques and advanced machine learning approaches in understanding the intricacies of neurodevelopmental disorders, opening the path for more effective diagnostic tools and deeper insights into the brain.

An Unsupervised Contrastive Graph Learning (UCGL) framework for resting-state fMRI research (8) underlines the difficulty of acquiring labelled training data in clinical practice and suggests a pretext model trained on unlabeled data for subsequent illness diagnosis tasks. The UCGL framework is tested on three rs-fMRI datasets, and it outperforms current techniques in the automated diagnosis of major depressive illness, ASD, and Alzheimer’s disease. ASD is diagnosed using structural (sMRI) and functional (fMRI) MRI modalities (9). To solve the low data availability, transfer learning is used in conjunction with four vision transformers and a 3D-CNN model. The investigations use several ways to generate data and extract slices from raw 3D sMRI and 4D fMRI images, yielding ground breaking findings. The brain disease categorization in resting-state functional magnetic resonance imaging (rs-fMRI) data study (10). Using multi-omics data, the team created eleven networks that depicted various facets of the brain. Kullback-Leibler divergence is used in their methodology, Graph convolution techniques are then applied to learn gene-disease connections. Functional magnetic resonance imaging (fMRI) data has been more important in neuroscience research recently for the identification of neurological illnesses and for the comprehension of cognitive processes. Many research has looked at various approaches to deal with issues such noise, limited sample numbers, and the requirement for interpretability that arise while processing fMRI data. Applying fMRI data to categorize neurological disorders or cognitive function has been the subject of several research. Using fMRI-derived brain graphs, GroupINN (11)—a grouping-based interpretable neural network—classifies cognitive performance well. Node grouping is included into the architecture of this model, which jointly learns these groups and extracts graph properties. Using resting-state fMRI time-series data, networks of long- and short-term memories (LSTMs) (12) can be used to directly diagnose autism spectrum disorders (ASD). They used the extensive, multi-site Autism Brain Imagery Data Exchange (ABIDE) I database for testing and training, and as a result, their classification accuracy was higher than that of earlier techniques. By applying algorithms that use deep learning to the ABIDE dataset (13), it was possible to identify ASD patients only by looking at patterns of brain activation. This approach achieved a remarkable degree of accuracy and revealed functional connectivity patterns linked to ASD. ASD-DiagNet (14), a system for ASD identification using fMRI data, was developed utilizing the 1,035 participants from 17 imaging facilities in the Autism Brain Imaging Information Exchange dataset. Techniques include a hybrid strategy for feature extraction that uses a single layer the perceptron and an autoencoder, as well as a linear interpolation-based data augmentation technique. In order to overcome the shortcomings of conventional behavioral observation-based diagnostic techniques, the research emphasizes the importance of developing machine learning infrastructures for qualitative diagnosis of varied mental disorders like autism spectrum disorder.

In these studies, the ABIDE dataset has become a notable resource offering a big, multi-site collection of fMRI data for research on ASD. By using this dataset, researchers may test and train their models on a variety of samples, which improves the generalizability of their conclusions. This research uses a range of methodologies, from deep learning architectures to conventional machine learning approaches. While deep learning models frequently give better performance but lack transparency, classical models could offer interpretability. Thus, by putting forth models which are both practical and understandable, recent initiatives have sought to close this gap.

### 2.2 GNN-based ASD recognition

Adversarial Graph Contrastive Learning (A-GCL) (15) is a method for identifying neurodevelopmental problems using fMRI data. The model employs a graph neural network (GNN) based on graph contrastive learning, with graphs generated from fMRI data. A-GCL outperforms three datasets, Autism Brain Imaging Data Exchange (ABIDE) I, ABIDE II, and attention deficit hyperactivity disorder (ADHD), over three atlases. While an A-GCL model may provide a good theoretical fit, this may be underpinned with several limitations including high-quality data in fMRI, inability to generalize across very diverse populations, low interpretability, and computing demands. Specifically, the atlases used need to be determined and only the resting-state fMRI is adopted. A Multi-Scale Dynamic Graph Learning (MDGL) (16) framework for detecting brain disorders utilizing resting-state functional magnetic resonance imaging (rs-fMRI) data. They use various brain atlases to build multi-scale dynamic functional connectivity networks (FCNs) and graph neural networks to extract spatiotemporal information from them. ASD categorization based on brain functional activity and gene expression using an attention-based graph neural network (GNN) (17). Their findings highlighted the significance of customized information in ASD diagnosis and biomarker identification. By combining individual brain topology and graph data. Furthermore, they identified brain regions important for ASD, such as the social-brain circuit and default-mode network, and discovered ASD-related genes using functional MRI data and gene expression analysis, demonstrating the potential of their approach for effective ASD diagnosis and biomarker identification. Present the Autism Spectrum Disorder-based Attention GNN and Crossover Boosted Meerkat Optimization (ASD-AttGCBMO) (18) algorithm. The suggested technique uses structural Magnetic Resonance Imaging (sMRI) data from the ABIDE 1 dataset for preprocessing to improve picture quality. Surface-based analysis and voxel-based morphometry (VBM) both extract significant information such surface area, cortical thickness, shape descriptors, and brain volumes. To address issues such as overfitting and class imbalance, the model utilizes attention GNNs with crossover-boosted meerkat optimization. PLSNet (19) is a position-aware graph-convolution-network-based model for ASD diagnosis that uses functional MRI (fMRI) data. PLSNet includes a time-series encoder for feature extraction and a connection generator to represent long-term relationships. PLSNet includes a time-series encoder for feature extraction and a connection generator to represent long-term relationships. Position embedding and a rarefying approach are used to solve challenges such as brain region variation and dimensionality complexity. The work delivers cutting-edge performance on the Autism Brain Imaging Data Exchange dataset.

The neuroimaging methods, namely magnetic resonance imaging (MRI) and functional MRI (fMRI), to diagnose Autism Spectrum Disorder (ASD). Each research suggests a unique methodology, such as machine learning techniques, generative adversarial networks (GANs), unsupervised contrasting graph learning, and adversarial self-supervised GNNs. A Conditional Generative Adversarial Network (cGAN) for predicting ASD (20). The researchers emphasize the limits of classic supervised machine learning techniques when dealing with tiny datasets, and they provide a cGAN that surpasses normal GANs in terms of prediction accuracy. A graph attention network (GAT) (21), based on spatially restricted sparse functional brain networks (FBNs), was used to diagnose ASD. They developed a unique approach, Pearson’s correlation-based Spatial Constraints Representation (PSCR), for estimating FBN structures and feeding them into a GAT for classification. Their trials using the ABIDE I dataset demonstrated the superiority of the PSCR technique as well as the influence of various FBNs on classification outcomes. Their suggested system produced encouraging classification results, surpassing rival approaches and offering insights for future illness detection using FBN and GNN frameworks.

The focus is on ASD, and task-based functional magnetic resonance imaging (fMRI) data is used. The authors focus on data-driven learning algorithms for biomarker identification and outcome prediction. Their deep learning methods use GNNs (GNNs) (22) for spatial variables and Long Short-Term Memory (LSTM) networks for temporal features. The chapter emphasizes the significance of dynamic connectivity changes and provides a more comprehensive, integrated model that includes spatiotemporal aspects as well as neural ordinary differential equations. ASD (23), utilizing a thorough examination of multi-modal imaging markers. Their dual-branch GNN performs a major diagnosis by extracting and combining data from structural and functional magnetic resonance imaging. The study also uses a perturbation model to find brain imaging signals and a neuro-transcriptomic joint analysis to reveal putative genetic biomarkers related with ASD brain development. Contribute to psychiatric diagnosis (24) using brain-networks by presenting a Granger causality-inspired GNN (CI-GNN). The model strives for interpretability without resorting to *post-hoc* interpretative paradigms.

A graph neural network architecture called BrainGNN (25) was used to analyze fMRI datasets from the Human Connectome Program (HCP) 900 Participant Release and Autism Spectrum Disorder (ASD). Their solution leverages both functional and topological knowledge gathered from fMRI data by using new ROI-aware graph convolution layers (Ra-GConv). Furthermore, BrainGNN uses ROI-selection layer pooling (R-pool) to emphasize important brain areas, making it easier to read. Regularization terms are suggested to promote flexible modeling of single or group-level patterns and fair ROI selection, such as units loss, topK pooled (TPK), which loss, and group-level constancy (GLC) loss.

### 2.3 GCN-based ASD recognition

In recent research, there has been an increasing emphasis on using functional brain networks (FBN) to classify neurological illnesses, particularly Autism Spectrum Disorder (ASD). MVS-GCN (2), a multi-view graph convolution network guided by previous brain structure learning, to overcome the issues presented by subject heterogeneity and noise correlations in brain networks. Their machine learning technique not only helps to classify neurological illnesses, but also gives an interpretable framework for deeper insights into the brain network. A joint learning architecture of multi-level dynamic brain networks for the diagnosis of ASD (26). They overcome the constraints of previous graph convolutional network (GCN)-based techniques by allowing bidirectional information sharing across brain networks and adding edge weight information via an edge self-attention mechanism. It allows for information complementarity across different layers of brain networks. The Autism Spectrum Disorder-based Attention Graph Neural Network and Crossover Boosted Meerkat Optimization (ASD-AttGCBMO) method (27) detects ASD using structural MRI data. Their approach uses attention graph neural networks and a crossover boosted optimization strategy to improve feature categorization between ASD and control participants. Graph Neural Network (GNN) topologies and machine learning models for analyzing rs-fMRI data to better understand schizophrenia (28). They train deep graph convolutional neural networks (DGCNNs) and machine learning models with graph-theoretical measurements based on functional correlations between brain areas of interest CI-GNN finds influential subgraphs associated with choices (e.g., major depressive disorder), meeting the demand for interpretable graph neural networks and highlighting the significance of causal linkages in explainability. Moving on to neurodegenerative illnesses (29), investigates early dementia prediction using fMRI data and a Graph Convolutional Network (GCN) technique A multi-task learning strategy using a knowledge graph attention network to identify both mental and physical diseases (MPD) (30) simultaneously.

Investigating disparities in brain activity to differentiate between individuals with Autism Spectrum Disorder (ASD) and those without aids in understanding the root causes of ASD, leading to enhanced diagnosis and treatment strategies. As a result, functional connectivity (FC) analysis (31) derived from resting-state functional magnetic resonance imaging (rs-fMRI) data has emerged as a potent method for assessing and charting brain activity.

Using a supervised the siamese graph convolution neural network (s-GCN) (32) as a foundation, this approach learns a graph similarity measure with a specific emphasis on comparing brain connection networks. The model takes graph structure into account by using spectral graph convolutions, which function in the graph spectral domain. This improves results on the ABIDE database. Graph neural networks, with applications in neurology and other domains, have shown promise as a method for processing graph-structured data. During the graph representation learning process, local structural information is preserved by the use of EigenPooling (33), a pooling operation based on the graph Fourier transform. By fusing layers for pooling based on EigenPooling with conventional graph convolutional layers, they create EigenGCN, a graph neural network architecture for graph classification, and demonstrate its efficacy on six widely used benchmarks. To overcome the shortcomings of current approaches in the field of resting-state functional magnetic resonance imaging (rs-fMRI) (34) research, formulate functionally connected networks as spatiotemporal graphs. To simulate the non-stationary character of functional connectivity, they present a spatio-temporal graphing convolutional network (ST-GCN) built on brief sub sequences of BOLD time series. When it comes to predicting age and gender from BOLD data, ST-GCN performs better than standard methods. It also finds key brain areas and functional linkages that are involved in the predictions.

Functional brain networks (FBN) have attracted a lot of interest as a means of diagnosing neurological disorders including autism spectrum disorders (ASD). Accurate categorization is difficult due to noisy correlations in brain networks and considerable subject heterogeneity. MVS-GCN (2), which combines graph neural networks to get efficient end-to-end representations for brain networks. To improve classification performance and find possible functional subnetworks, this approach combines multi-view graph convolutional neural networks with previous knowledge of brain anatomy. Using the Alzheimer’s Disease Neuroimaging Initiative (ADNI) and Autism Brain Imaging Data Exchange (ABIDE) datasets, the authors assess the MVS-GCN model and show that it is more effective than current techniques. This concept motivates to generate a integrating the views in a combined form with the help of transformer and introduced a new Multi-View United Transformer Block (MVUTB). The proposed model also improves the performance of class discrimination using Graph Attention Network.

## 3 Background

### 3.1 Graph attention layer on ASD

The Graph Attention Layer (GAT) is critical in identifying essential patterns and characteristics in neuroimaging data for recognizing Autism Spectrum Disorder (ASD). The application of GAT is discussed here, with equations to show how it works in the context of ASD recognition.

The GAT layer in the ASD identification task receives a collection of node characteristics reflecting neuroimaging data connected with brain areas (34). Let $h=\left\{{\hat{h}}_{1},{\hat{h}}_{2},\ldots ,{\hat{h}}_{N}\right\}$ N is the number of brain regions, and F is the number of related characteristics for each area. The goal is to create a new set of node functionalities $\;h_{0}=\left\{{\hat{h}}_{01},{\hat{h}}_{02},\ldots ,{\hat{h}}_{0N}\right\}$ , with ${\hat{h}}_{01}in\;{\mathbb{R}}^{F_{o}}$ as Output.

The transformation starts with a common linear transformation, parameterized by a weight matrix. *W* in ${\mathbb{R}}^{F_{o}X\;F}$ , applied to each node’s features:


(1) $$\hat{h}{'}_{i}=W{\hat{h}}_{i}$$

A self-attention mechanism is used to produce attention coefficients (*e_ij_*) that indicate the significance of characteristics from node j to node i for ASD detection. The attention coefficients are generated using a shared attentional mechanism and masked attention, focusing solely on first-order neighbors.


(2) $$e_{ij}=a\left(\;{\hat{h}}_{i}^{'}{\;}_{i},{\hat{h}}_{j}^{'}{\;}_{j}\right)$$

To introduce the graph structure, masked attention is conducted, only for the node **j** which is a neighbor of node i, that is taking just the neighborhood *N_i_* of node i. To make coefficients comparable across nodes, a softmax function is applied:


(3) $$a_{ij=Softmax\left(\frac{exp\left(e_{ij}\right)}{\sum_{k\in N_{i}}exp\left(e_{ik}\right)}\right)}$$

Finally, the new node includes ${\hat{h}}_{0i}$ is calculated as a weighted sum of the original characteristics, using attention coefficients as weights.


(4) $${\hat{\text{h}}}_{0i}=\sum_{j\in N_{i}}a_{ij}\hat{\text{h}}{'}_{j}$$

This method efficiently captures the significance of characteristics from surrounding brain areas in the context of ASD identification (35) by utilizing the self-attention mechanism and graph structure information. The GAT layer’s capacity to selectively aggregate information depending on attention coefficients improves the overall efficacy of ASD detection in neuroimaging data.

### 3.2 Transformer-based ASD recognition

Transformer-based models have gained popularity in recent neuroimaging studies for detecting Autism Spectrum Disorder (ASD) (36, 37). Transformers, which were first introduced for natural language processing, have shown amazing ability to capture subtle patterns and relationships within sequential data, making them ideal for analyzing complex brain imaging data linked with ASD.

#### 3.2.1 Overview of transformer model

The Transformer design includes an encoder-decoder structure, however in the context of ASD identification, we concentrate on the encoder. The Transformer encoder’s main components include self-attention mechanisms (38), multi-head self-attention (MHA), and feedforward neural networks (FFN).

#### 3.2.2 Self-attention mechanisms

The self-attention mechanism is a critical component of the Transformer model, allowing it to weigh various elements of the input sequence independently. Given an input sequence, the self-attention mechanism calculates attention ratings for each element in the sequence in comparison to all other items. The attention scores are then utilized to calculate a weighted total of the input sequence, which results in context-aware representations for each piece. The mathematical expression for the self-attention process is as follows:


(5) $$Attention\left(Q,K,V\right)=Softmax\left(\frac{QK^{T}}{\sqrt{d_{k}}}\right)V\;$$

The query, key, and value matrices are represented by *Q, K, V*, respectively, and ***d_k_*** denotes the dimension of the key vectors.

#### 3.2.3 Multiple-head self-attention

To increase model expressiveness, the Transformer utilizes many attention heads in tandem. Each head gains a separate understanding of the relationships within the input sequence. The outputs from the several heads are concatenated and linearly processed to generate the final attention output. The formula for multi-head self-attention is as follows:


(6) $$MHA\left(Q,K,V\right)=Concat\left(Head_{1},\ldots \ldots ,Head_{n}\right)W_{o}$$

Where $Head_{i}=Attention\left(QW_{Qi},KW_{Ki},VW_{Vi}\right)$ and $W_{o}\;$ ​ is the output projection matrix.

#### 3.2.4 Feedforward neural network

After the attention process, the model uses feedforward neural networks to perform further nonlinear transformations. The FFN is made up of two linear layers separated by a non-linear activation function (often a ReLU). The mathematical expression for the FFN operation is as follows:


(7) FFN(x)=ReLU(xW1+b1)W2+b2

The weight matrices and bias terms are represented by  W1, b1, W2 and b2 .

In the context of ASD detection, the Transformer encoder examines neuroimaging data, such as MRI scans, to identify ASD-related characteristics and patterns. The self-attention mechanism allows the model to focus on specific areas of interest, whilst the multi-head mechanism improves the model’s capacity to collect varied and complicated correlations in brain data. The ensuing feed forward neural networks modify the representations for downstream tasks, such as distinguishing between ASD and control participants.

## 4 Proposed model of ASD multi-view united transformer block

In this section, we introduce the ASD Multi-View United Transformer Block (ASD-MVUTB), a new model for detecting Autism Spectrum Disorder (ASD). Our suggested design uses transformer blocks to combine multi-view information, taking use of varied views from neuroimaging data. The model effectively captures subtle patterns from several perspectives, encouraging complete feature extraction for accurate ASD detection. Experimental validation shows that the proposed ASD-MVUTB outperforms existing techniques.

**Figure 1:**
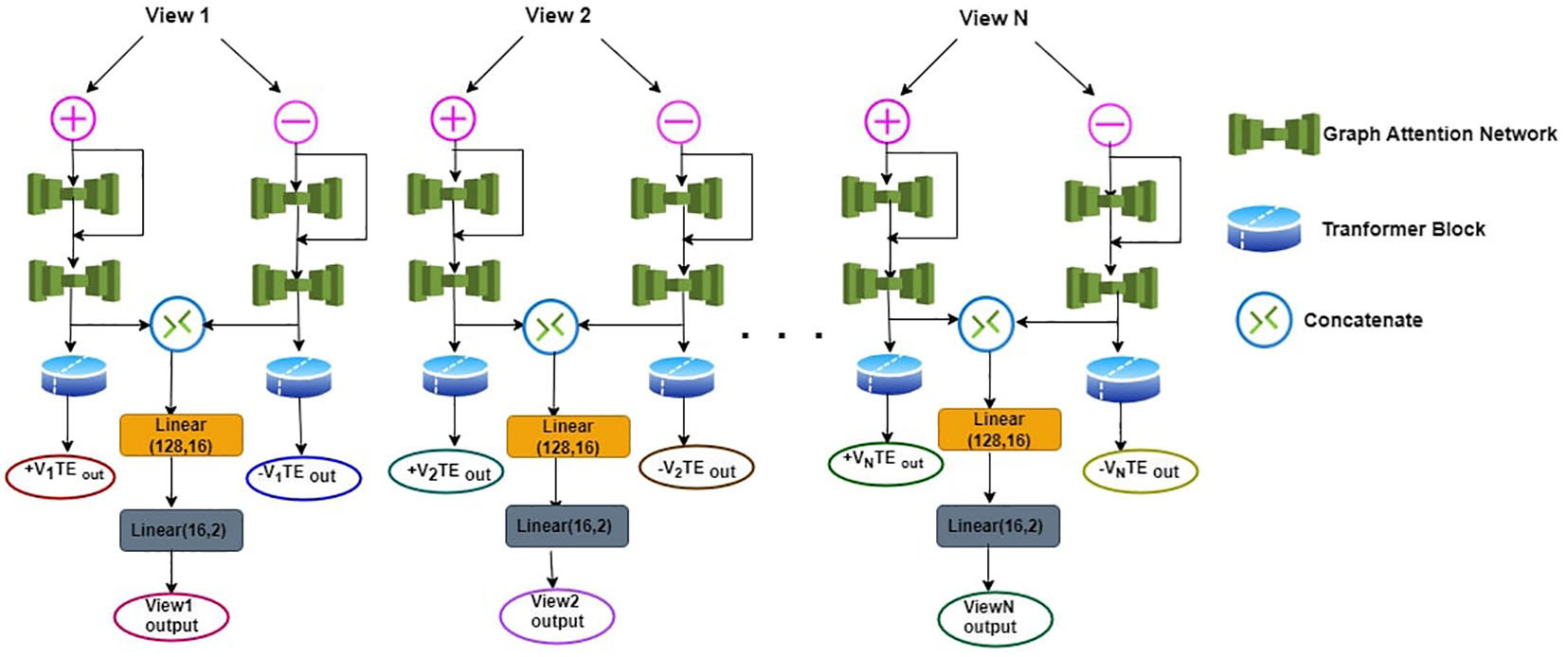
Workflow of detecting ASD.

The workflow for detecting Autism Spectrum Disorder (ASD) in **Figure 1**, is structured around N distinct views (V_1_, V_2_, and V_N_), each comprising both positive (+V) and negative (-V) perspectives. In the V_1_-Positive View (+V), the process initiates with a Graph Attention Network (GAT-1), wherein neuroimaging data undergoes attention-based feature extraction. The output of GAT-1 is then subject to a Residual Addition operation, combining the GAT-1 output and the original positive view data. Subsequently, another Graph Attention Network (GAT-2) is applied, repeating the attention-based feature extraction process. The results of GAT-2 for both +V and -V are concatenated, and the concatenated output undergoes two linear layers (linear (128,16) and linear (16,2)) to determine the binary classification output, indicating whether the patient exhibits ASD (39, 40).

Mathematically, the process can be expressed as follows:

1. GAT-1 Process:


(8) GAT−1(+V)=GAT−1 Output

2. Residual Addition:


(9) GAT−1 Output++V=Residual Addition

3. GAT-2 Process:


(10) GAT−2(Residual Addition Output)=GAT−2 Output

4. GAT-2 Layer Concatenation:


(11) Concatenate(GAT−2 Output (+V), GAT−2 Output (−V))

5. Linear Layer Application:


(12) $$\text{Linear }(128,\;16)\to \text{Lineary }(16,\;2)=\text{V}\text{i}\text{e}{\text{w}}_{1}\text{ Output}$$

Next, after the GAT-2 process for each view, a Transformer Encoder (TE) is applied. The TE operation is conducted separately for both the +V and -V outcomes of each view. This multi-view approach enhances the model’s ability to capture diverse features and patterns related to ASD within the neuroimaging data.

The Transformer Encoder for all View (V’s) is expressed as:


(13) TE(GAT−2 Output(+V))=+V TE Output

(14) TE(GAT−2 Output(−V))=−V TE Output

This entire process is replicated for View_2_ (V_2_), View_3_ (V_3_) and upto View_N_(V_N_). In this work three views are used to construct the ASD model. The utilization of Graph Attention Networks and Transformer Encoders facilitates capturing intricate patterns in neuroimaging data, and the linear layers aid in synthesizing and classifying the information for ASD detection. The concatenated results from the GAT-2 process ensure a comprehensive representation of both positive and negative perspectives in each view, contributing to the overall diagnostic outcome.

**Figure 2:**
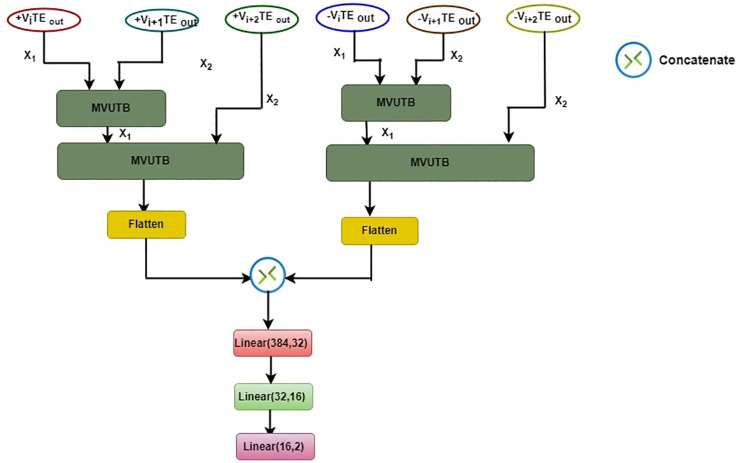
ASD-MVUTB proposed model architecture.

The proposed ASD-MVUTB model architecture, as illustrated in **Figure 2**, involves distinct processing of positive and negative TE outputs from Views (+Vs and -Vs) separately. Specifically, the TE outputs +V_i_TEout (X1) and +V_i+1_TEout (X2) are processed independently in the first MVUTB layer. The resulting outputs, denoted as X1 and X2, are then combined with +V_i+2_TEout in the second MVUTB layer. These intermediate results are flattened to create feature vectors. A parallel process is applied to the negative TE outputs, resulting in three sets of flattened feature vectors. Subsequently, the flattened results from both +Vs and -Vs are concatenated, and the concatenated feature vector undergoes three linear transformations: Linear(384, 32), Linear(32, 16), and Linear(16, 2).

The ASD-MVUTB working process is represented as follows:

1. MVUTB layers:

• Positive TE Outputs Processing.


(15) $$X1=MVUTB\left(+V_{i}TE_{OUT},\;+V_{i+1}TE_{OUT}\right)$$

(16) $$X2=MVUTB\left(X1,\;+V_{i+2}TE_{OUT}\right)$$

• Negative TE Outputs Processing.


(17) $$Y1=MVUTB\left(-V_{i}TE_{OUT},\;-V_{i+1}TE_{OUT}\right)\;$$

(18) $$Y2=MVUTB\left(Y1,\;-V_{i+2}TE_{OUT}\right)$$

2. +Vs and –Vs Flatteninng.


(19) $$F_{X}=Flatten\left(X1,\;X2\right)$$

(20) $$F_{Y}=Flatten\left(Y1,\;Y2\right)$$

3. Concatenation:


(21) $$F_{Concat}=Concat\left(F_{X},\;F_{Y}\right)$$

4. Linear Transformations:


(22) $$Z1=Linear\;\left(F_{Concat},\;384,32\right)$$

(23) Z2=Linear (Z1,32,16)

(24) Z3=Linear (Z2, 16, 2)

The motivation behind this architecture lies in the enhanced feature extraction from both positive and negate+ve TE outputs independently, and the subsequent concatenation allows the model to capture and leverage information from both perspectives. The linear layers at the end serve to further distill and map the concatenated features into a final prediction space for ASD classification. This layered approach enhances the model’s ability to discern intricate patterns and relationships within the TE outputs, contributing to its effectiveness in ASD recognition.

**Figure 3:**
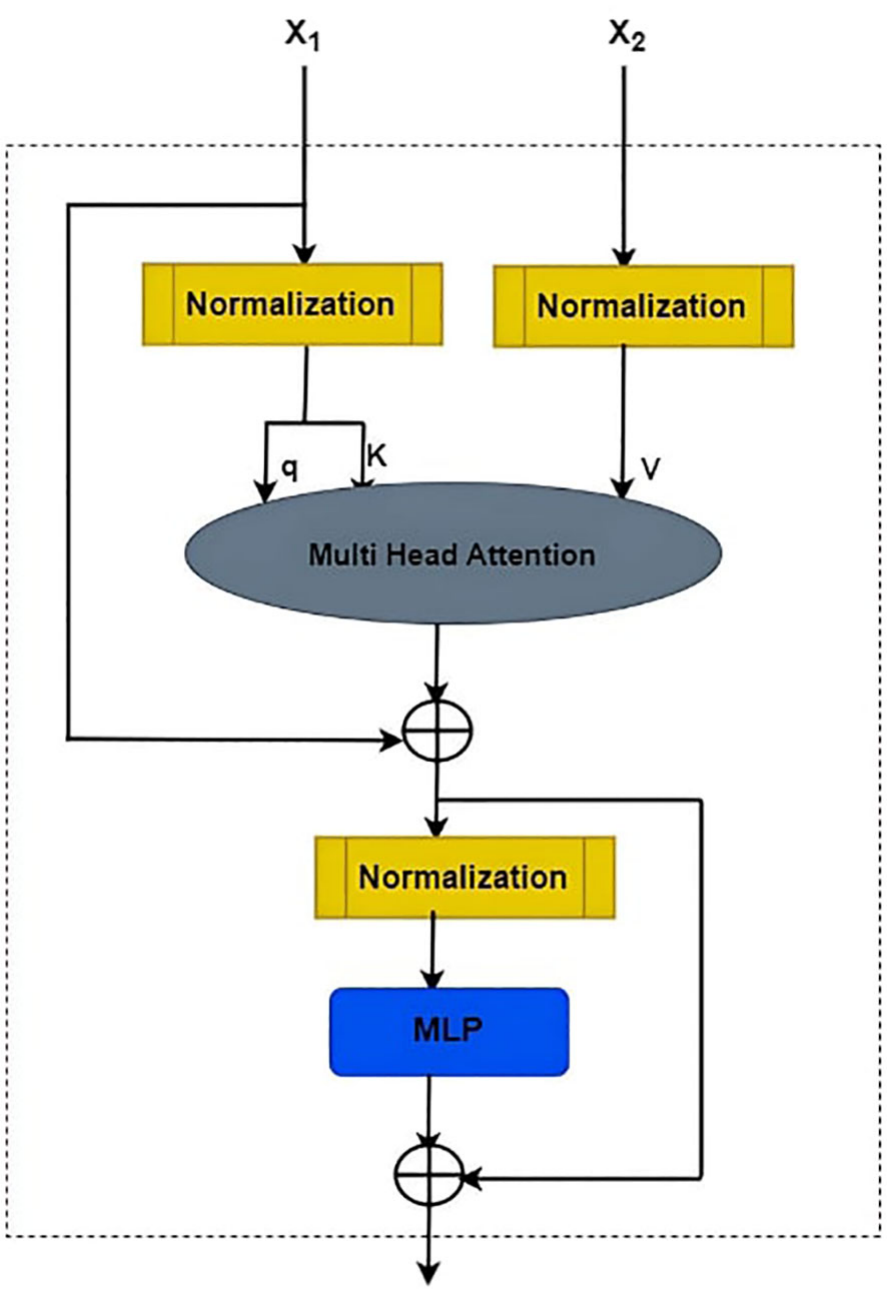
Framework of multi-views in transformer block.

In the proposed framework depicted in **Figure 3**, the Multi-View Transformer Block operates through a meticulous process involving two views of features, denoted as X1 and X2. These views undergo Layer Normalization simultaneously. Subsequently, a linear mapping is applied to the query (q) and key (k) from X1, and the value (v) from X2, which are then fed into the Multi-Head Attention (MHA) mechanism. The output of MHA, along with the original features from X1, undergoes an element-wise addition (A1) to yield a preliminary result (R1). Following this, another Layer Normalization is applied, succeeded by a Multi-Layer Perceptron (MLP). The output of the MLP, combined with the result from the previous addition (R1), undergoes a second addition (A2), culminating in the generation of the final outcome. This iterative and additive process allows for the effective integration of information from multiple views.

1. Layer Normalization:


(25) $$LayerNorm\left(X\right)=\frac{\left(X-\mu \right)}{\sqrt{\sigma^{2}+\epsilon }}\times \;\gamma +\beta$$

Where *μ* and *σ* represent the mean and standard deviation, *ϵ* is a small constant, and *γ* and *β* are learnable parameters.

2. Multi-Head Attention (MHA):


(26) $$MHA=\left(q_{x1},k_{x1},V_{x2}\right)=Softmax\;\left(\frac{q_{x1},\;k_{x1}^{T}\;}{\sqrt{d_{k}}}\right)V_{x2}$$

3. Addition (A1):


(27) $$R1=X1+MHA\left(q_{x1},k_{x1},V_{x2}\right)$$

4. Multi-Layer Perceptron (MLP):


(28) $$MLP\left(X\right)=ReLU\left(XW_{1}+\;b_{1}\right)W_{2}+b_{2}$$

5. Addition (A2):


(29) R2=R1+MLP(R1)

The adoption of Multi-Head Attention allows the model to capture intricate relationships within and between X1 and X2, enhancing representational capacity. The subsequent addition of the MHA output and the original X1 features promotes the fusion of learned contextual information with the original content. The Layer Normalization following each operation ensures stability and normalization of the intermediate results. The introduction of the MLP introduces non-linear transformations, further enhancing the model’s ability to capture complex patterns. The final addition consolidates the MLP’s output with the result from the initial MHA addition, producing a comprehensive and refined representation that incorporates information from both views. This iterative process aligns with the principles of transformer-based architectures, facilitating multi-view feature integration for improved learning and representation.

## 5 Experimental results

The experimental findings in this section show how well the Multi-View United Transformer Block of the Graph Attention Network detects ASD. Using a variety of neuroimaging modalities, including as MRI and fMRI data, the model performs exceptionally well in identifying subtle patterns linked to ASD. In addition, the inclusion of attention processes improves interpretability and makes it possible to find significant biomarkers. These findings highlight the potential of deep learning techniques to transform the knowledge and early identification of ASD.

In **Table 1**, a comprehensive comparison between the proposed Multi-View United Transformer Block of Graph Attention Network (MVUT_GAT) and several existing methods reveals notable advancements in accuracy. Compared to the MVS-GCN (1) approach the MVUT_GAT exhibits a superior performance, surpassing MVS-GCN by +3.4%, ASD-DiagNet by +4.98%, and DAE by +5.68%. These substantial accuracy improvements underscore the efficacy of the proposed method in outperforming various state-of-the-art approaches in Autism Spectrum Disorder identification.

**Table 1:** Comparison of existing methods for sensitivity, specificity, accuracy, and AUC.

	Accuracy	Sensitivity	Specificity
DAE (12)	67.61	78.7	53.2
ASD-DiagNet (13)	68.31	60.31	67.76
GroupINN (10)	63.6	61.52	57.36
ST-GCN (33)	57.29	54.78	48.91
Eigenpooling GCN (32)	57.5	58.81	59.94
LSTM-ASD (11)	68.5	-	-
BrainGNN (24)	61.84	61.65	60.79
sGCN (31)	67.54	64.73	60.12
MVS-GCN (1)	69.89	70.18	63.05
MVUT_GAT	73.29	73.86	69.67

- means sensitivity and specificity are not applicable.

**Figure 4:**
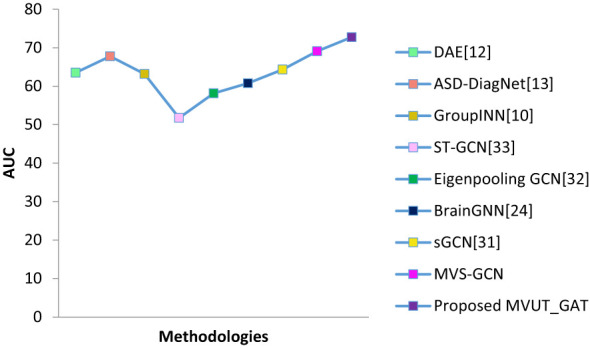
The comparison of various methods AUC on the ABIDE dataset.

A comparative examination of the Area Under the Curve (AUC) values for several approaches on the ABIDE dataset is shown in **Figure 4**. An extensive summary of each technique’s efficacy in diagnosing Autism Spectrum Disorder (ASD) is given by the AUC, a statistic used to evaluate the performance of classification models. The graph provides information about the relative advantages and disadvantages of the models by graphically representing the discriminative capacity of different methodologies. The most promising techniques for precise ASD diagnosis are chosen by academics and clinicians with the help of this quantitative assessment based on the ABIDE dataset. When evaluating how well various algorithms handle neuroimaging data for ASD identification, the figure is a useful point of comparison.

**Figure 5:**
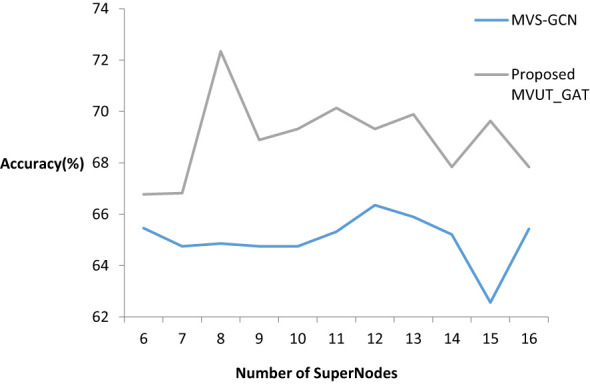
Performance for different number of super nodes of coarsened graphs.

**Figure 5** displays the values of the super node number for the graph structure learning and the corresponding outcomes. Findings indicate that the number of nodes in the coarsened graph significantly affects classification performance, indicating the importance of the brain network’s coarsening level for graph structure learning. More specifically, the coarsened graph’s topology information is condensed if there are fewer super nodes than there are, and richer if there are more super nodes than there are.

**Figure 6:**
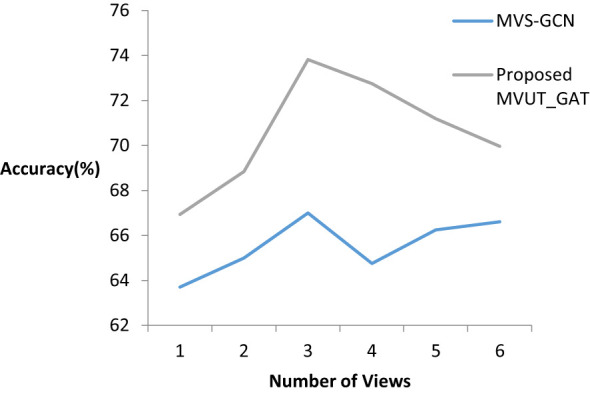
Performance variation as the number of views increases.

V ∈ {1, 2, 3, 4, 5, 6} was changed, and the performance variation with different values was examined in order to examine the impact of variability throughout the number of views. As seen in **Figure 6**, the performance gets better the more views there are, up to a maximum of three. This indicates that distinct views have built-in correlations that help capture topological details. Further evidence that more views will unavoidably contribute duplicate data to the model and lower its classification performance comes from the observation that the model’s performance tends to stabilize as the number of views rises.

## 6 Conclusion and discussion

Identification of Autism Spectrum Disorder (ASD) may advance with the incorporation of deep learning models, such as the Multi-View United Transformer Block of Graph Attention Network. These algorithms’ capacity to automatically identify subtle abnormalities in multimodal neuroimaging data improves diagnostic precision and makes it easier to identify ASD early on. By including attention processes, interpretability is further enhanced and the gap between clinically applicable discoveries and complicated models is closed. The proposed model Multi-View United Transformer Block of Graph Attention Network (MVUT_GAT) outperforms the existing model by +3.4% which improves the robustness and generalizability of ASD diagnostic models. In order to improve the robustness and generalizability of ASD diagnostic models, continued research efforts should concentrate on honing architectures, optimizing models, and investigating hybrid techniques as deep learning continues to develop. To further enhance our knowledge of the neurological foundations of ASD, future research may investigate the integration of other data sources, including as genetic markers and behavioral assessments. These developments might transform early diagnosis and intervention approaches, ultimately leading to better outcomes for people with ASD worldwide.

## 7 Future work

Future studies on MVUT_GAT for ASD diagnosis will focus on some key issues to improve the robustness and generalizability of the model. Among these, the main focuses should be on optimizing the model, hyperparameters refinement, an increase in efficiency levels along with transfer learning, and other advanced techniques that can help it handle such datasets. Additionally, MVUT_GAT could be combined with other deep learning models, like CNNs or RNNs, to enhance performance. Multimodal data integration, such as genetic markers, behavioral assessments, and neuroimaging, would give a better understanding of ASD and could also enhance diagnostic accuracy. Improving interpretability is yet another important area. Mechanisms of attention and explainability techniques, such as the saliency maps used by the model, will clarify the decision-making process for clinicians, so they can better trust and implement deep learning models in practice while filling a gap between advanced algorithms and clinical use. Longitudinal studies should be pursued to track the changes in ASD over time with neuroimaging data. This will ensure earlier detection of ASD and real-time tracking of interventions. A greater population from more diverse demographics would increase the number of datasets, which would be more generalizable for the model and its applications in different settings of healthcare. Finally, collaboration among deep learning researchers, clinicians, and neuroscientists will be very critical to align the models with clinical needs and ensure that these advances are translated into better ASD diagnosis and intervention, hence improving outcomes for individuals around the world.

## Data Availability

Publicly available datasets were analyzed in this study. This data can be found here: https://fcon_1000.projects.nitrc.org/indi/abide/.
